# Characterization of parasporin gene harboring Indian isolates of *Bacillus thuringiensis*

**DOI:** 10.1007/s13205-013-0190-9

**Published:** 2013-12-17

**Authors:** N. K. Lenina, A. Naveenkumar, A. E. Sozhavendan, N. Balakrishnan, V. Balasubramani, V. Udayasuriyan

**Affiliations:** Department of Plant Biotechnology, Centre for Plant Molecular Biology and Biotechnology, Tamil Nadu Agricultural University, Coimbatore, 641003 India

**Keywords:** *Bacillus thuringiensis*, Parasporin, δ-endotoxin, Non-insecticidal inclusions, Cytocidal protein

## Abstract

*Bacillus thuringiensis* (Bt) is popularly known as insecticidal bacterium. However, non-insecticidal Bt strains are more extensively available in natural environment than the insecticidal ones. Parasporin (PS) is a collection of genealogically heterogeneous Cry proteins synthesized in non-insecticidal isolates of Bt. An important character generally related with PS proteins is their strong cytocidal activity preferentially on human cancer cells of various origins. Identification and characterization of novel parasporin protein which are non-hemolytic and non-insecticidal but having selective anticancer activity raise the possibility of a novel application of Bt in medical field. In the present study, seven new indigenous isolates (T6, T37, T68, T98, T165, T186, and T461) of Bt showed variation in colony morphology, crystal characters and protein profiles with each other. Out of the seven new isolates screened for parasporin (*ps*) and *cry* genes, two of the new indigenous isolates (T98 and T186) of Bt showed the presence of *ps4* gene. Partial *ps4* gene was cloned from the two new isolates and the sequence of partial *ps4* gene showed high homology with its holotype *ps4Aa1.* These two isolates were characterized based on the proteolytic processing of the inclusion proteins and the proteolytic products were found to be comparable to the PS4 reference strain A1470. The two isolates of Bt did not show toxicity toward *Spodoptera litura* and *Helicoverpa armigera.* Based on the results of this study, it can be concluded that the isolates T98 and T186 are parasporin producers.

## Introduction

*Bacillus thuringiensis* (Bt) is an aerobic gram-positive and endospore-forming bacterium, first isolated in Japan from diseased larvae of the silkworm, *Bombyx mori*, as an entomopathogenic bacterium (Ishiwata 1901). It produces large crystalline parasporal inclusions in sporangia during sporulation (stationary phase of its growth cycle). This character is used to discriminate two taxonomically closely related species, *B. thuringiensis* and *B. cereus* (Logan 2005; Ohba et al. 2009). The parasporal inclusions often contain δ-endotoxin proteins that are specifically toxic to agriculturally and medically important insect pests of several orders, including Lepidoptera, Diptera, and Coleoptera (Beegle and Yamamoto 1992) and to even nematodes, mites, and protozoa (de Maagd et al. 2001), but are not pathogenic to mammals, birds, amphibians, or reptiles (http://www.lifesci.sussex.ac.uk/home/Neil_Crickmore/Bt/) (Schnepf et al. 1998). This makes *B. thuringiensis*, a promising microbial agent in the control of insect pests in agriculture, forestry, veterinary, and public health management (Schnepf et al. 1998).

Meanwhile, non-insecticidal *B. thuringiensis* strains are ubiquitous in natural environments and are more widely distributed than insecticidal ones (Ohba 1996). It is remarkable that the non-insecticidal isolates frequently account for more than 90 % of the natural populations from soils (Ohba et al. 2002; Yasutake et al. 2007; Mizuki et al. 1999a, b). This raises the query whether non-insecticidal inclusions have any biological activity which is yet to be undiscovered (Ohba et al. 1988). An extensive effort to screen Cry proteins for biological activity other than insecticidal toxicity was initiated in 1996. This led to the discovery of a unique activity, which is preferential for certain human cancer cells (Mizuki et al. 1999a). The protein was first categorized and defined as bacterial parasporal proteins and these proteins are non-hemolytic but cytocidal to human cancer cells (Mizuki et al. 1999a, 2000). Globally, six different parasporin types, PS1–PS6 have been identified in countries, viz. Japan, Vietnam, India, Canada, and Caribbean Islands (Gonzalez et al. 2011) and classified by the Committee of Parasporin Classification and Nomenclature (http://parasporin.fitc.pref.fukuoka.jp/list.html). In view of potential application of these proteins, this study was undertaken to characterize new isolates of Bt collected from Western Ghats, India, based on colony and crystal morphology, protein profile, screening for presence of *cry* or *parasporin* genes by PCR and insect bioassay.

## Materials and methods

### Bacterial strains and plasmids

The bacterial strains used in this study were *B. thuringiensis* soil isolates (T6, T37, T68, T98, T165, T186, and T461) from Western Ghats of Tamil Nadu State, India (Ramalakshmi and Udayasuriyan 2010), and maintained in the Department of Plant Biotechnology, CPMB&B, Tamil Nadu Agricultural University, Coimbatore. The reference strains for parasporin (*ps*) genes, A1190 (*ps1*), A1547 (*ps2*), A1462 (*ps3*), and A1470 (*ps4*), provided by Dr. Natsuko Kurata, Biotechnology and Food Research Institute, Fukuoka Industrial Technology Centre, Japan, were used in this study. Bt strains, HD1 (*cry1* and *cry2*) and 4Q7 (acrystalliferous) were used as reference strains. *Escherichia coli* (DH5α) was used as a host for cloning the gene. The vector, pTZ57R/T (Fermentas Inc., Canada) was used to clone parasporin gene fragments amplified from new isolates of Bt. The antibiotic concentration used for selection of *E. coli* transformants was 100 μg/ml of ampicillin.

### Culture conditions

*Bacillus thuringiensis* culture was grown on T3 medium (Martin and Travers 1989) at 30 °C at 200 rpm for 2–8 days and the bacterial sporulation was monitored through phase contrast microscope for 2–8 days. *E. coli* was grown on LB medium for 24 h at 37 °C at 200 rpm.

### Characterization of isolates for colony and crystal morphology

The *B. thuringiensis* isolates streaked on T3 agar plates were incubated at 30 °C for 2–8 days. Colony morphology was studied on single colonies developed on T3 agar plates. The Bt isolates inoculated in 5 ml of T3 broth were incubated at 30 °C at 200 rpm for 2–8 days, and the bacterial sporulation was monitored through phase contrast microscope at 100×. After about 90 % of cell lysis, a smear of 10 μl lysed culture was made on glass slide and heat fixed. After heat fixing, drops of the Coomassie Brilliant Blue stain (0.133 % Coomassie Brilliant Blue G250 in 50 % acetic acid) were added and kept as such for 1 min. Then, the smear was washed gently in running tap water. After blot drying with blotting paper, the stained cultures were observed through bright field microscopy for presence of crystalline inclusions (Ramalakshmi and Udayasuriyan 2010).

### Preparation of inclusion proteins

The spore–crystal mixture was isolated from seven new isolates of Bt and reference strain A1470, as described by Lenin et al. (2001). Single colony of Bt strains was inoculated into 5 ml T3 broth and incubated in a rotary shaker, maintained at 30 °C at 200 rpm for 2–8 days, and the bacterial sporulation was monitored through phase contrast microscope. When more than 90 % of cells were lysed, the sporulated broth culture was transferred to 4 °C, at least half-an-hour before harvesting. The T3 broth containing spore–crystal mixture was centrifuged for 10 min at 10,000 rpm at 4 °C. The pellet was washed once with 5 ml of ice-cold 1× Tris–EDTA buffer [Tris 10 mM, EDTA 1 mM, pH 8.0 with 1 mM phenyl methyl sulphonyl fluoride (PMSF)], once with 5 ml of ice-cold 0.5 M NaCl followed by two more washes with 5 ml of Tris–EDTA buffer containing 1 mM PMSF by centrifuging at the same speed and time. Finally, the spore–crystal pellet was suspended in 100 μl of sterile distilled water containing 1 mM PMSF and stored at −20 °C.

### Screening of *parasporin* and *cry* genes

Screening of the test isolates for *ps* and *cry* genes was carried out in a 25-μl PCR reaction. Total genomic DNA isolated from Bt strains using Genei pure bacterial DNA purification kit (Genei, Bangalore, India) was used as template for PCR screening. The PCR was accomplished using an Eppendorf thermal cycler with a reaction mixture containing 50–100 ng of total genomic DNA of Bt, 1× PCR buffer (10 mM Tris–HCl; pH 9.0, 50 mM KCl, 1.5 mM MgCl_2_), 75 μM each of dNTPs, 50 ng each of forward and reverse primers (Table 1) and 1.5 U of *Taq* DNA polymerase.

**Table 1:** Primers used for screening of Bt isolates for different *cry* and *ps* genes

Primer sequences	Annealing °C	Gene	Amplicon size (bp)	Primer position in ORF		Reference
				FP	RP	
F: CATGATTCATGCGGCAGATAAAC	62	*cry1*	278	2,783	3,060	Ben-Dov et al. (1997)
R: TTGTGACACTTCTGCTTCCCATT						
F: GTTATTCTTAATGCAGATGAATGGG	64	*cry2*	702	570	1,271	Ben-Dov et al. (1997)
R: CGGATAAAATAATCTGGGAAATAGT						
F: ATCAAGAATTTTCCGATAATC	50	*ps1*	1,136	154	1,289	Yasutake et al. (2007)
R: CCAAAAGTGCCAGAATG						
F: TGTTGGGACTGTTCAGTACGT	56	*ps2*	503	341	843	*
R: CGTCACGGTACCTCTTAGTGT						
F: GGAATCCAGGTGCACTGCT	67	*ps3*	701	264	964	*
R: GTCCCGGATCATACGTTGGA						
F: AGTGGTCTCCAGGCTCATACTGG	59	*ps4*	681	81	761	*
R: TGATATTCCCGAACCTGCCCT						

* Designed in this study using Fast PCR 6.0

Template DNA was preheated at 94 °C for 2 min. Then it was denatured at 94 °C for 1 min, annealed to primers for 45 s and extensions of PCR products were achieved at 72 °C for 1 min. The PCR was performed for 30 cycles. The PCR products were analyzed on a 1.2 % agarose gel. Amplified product was ligated in pTZ57R/T PCR cloning kit and transformed into *E. coli* DH5α.

### Proteolytic processing of inclusion proteins

Spore–crystal mixture isolated from parasporin producing isolate was washed thrice with 1 M NaCl and resuspended in sterile water and transferred to a microfuge tube. After centrifugation at 13,000 rpm for 5 min at 4 °C, the pellet containing purified inclusions was solubilized in 50 mM Na_2_CO_3_ (pH 10.0) containing 1 mM EDTA and 10 mM dithiothreitol for 1 h at 37 °C (200 μl/25 mg pellet). After centrifugation at 13,000 rpm for 5 min at 4 °C, the supernatant was passed through 0.2-μm filter to remove unsolublized materials. The pH of the filtrate was adjusted to 8.0 and split into two equal aliquots. One of the aliquots of solubilized proteins was treated with proteinase K (final conc. 60 μg/ml), in 50 mM Na_2_CO_3_ (pH 10.0) for 90 min at 37 °C. After proteinase K treatment, 1 mM PMSF was added to the mixture to stop the proteolytic reaction. Both the aliquots (solubilized and proteinase K-treated inclusions) were subjected to sodium dodecyl sulfate-polyacrylamide gel electrophoresis (SDS-PAGE) analysis (Okumura et al. 2006; Saitoh et al. 2006).

### Toxicity analysis of new isolates

The laboratory cultures of *S. litura* and *H. armigera* reared on a semi-synthetic diet (Patel et al. 1968) were used to determine the insecticidal activity of the isolates T98 and T186 using diet surface contamination method. Approximately 1 ml of the semi-synthetic diet was dispensed into 1.8 ml cryovials (Tarson^®^; 1 cm dia.) and allowed to cool for an hour. After solidification of the diet, 10 μl spore–crystal mixture was coated on the diet surface and allowed to air dry for 30 min. Neonate larvae of *S. litura* and *H. armigera* were released using a soft hairbrush and the tube closed with a screw cap. All the above steps were carried out in a laminar airflow chamber. Vials without crystal mixture served as a control. Each treatment was replicated four times and ten vials were maintained for each replication. Larval mortality was recorded for 7 days and subjected to ANOVA. All the experiments were carried out in a room with a photoperiod of 14:10 (L:D) at an average temperature of 27 °C and 60 % relative humidity.

## Results and discussion

*Bacillus thuringiensis* formed white rough colonies which spread out and expanded over the plate quickly. Seven new isolates of Bt and six reference strains were observed for colony morphology on T3 plates. All the seven isolates produced creamy white colonies after 24 h of inoculation on T3 agar plates. The colony characteristics of test isolates showed slight variation with each other (Table 2). Chaterjee et al. (2006) also found similar variation in the morphological characteristics of Bt isolates on nutrient agar medium and reported circular, white, flat, and undulate colonies of the Bt isolates of West Bengal, India. The time taken for 90 % cell lysis in T3 broth was also observed for the new isolates along with the reference strains. The reference strains, HD1 and 4Q7 took 2–3 days, while the parasporin reference strains and the seven new isolates took 6–8 days for 90 % of cell lysis.

**Table 2:** Morphological characteristics of new isolates

Isolate	Color of colonies	Shape of colony	Margin of colony	Elevation of colony	Shape of inclusion
T6	Creamy white	Irregular	Undulated	Raised	Spherical
T37	Creamy white	Circular	Entire	Raised	Irregular
T68	Creamy white	Circular	Entire	Raised	Spherical
T98	Creamy white	Irregular	Undulated	Flat	Irregular
T165	Creamy white	Irregular	Undulated	Flat	Spherical
T186	Creamy white	Circular	Entire	Raised	Irregular
T461	Creamy white	Irregular	Undulated	Raised	Spherical

**Fig. 1:**
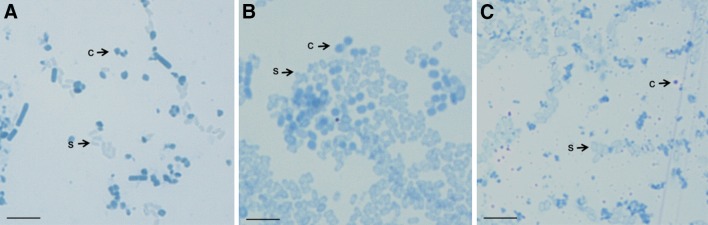
Bright field microscopic observation of crystal morphology from parasporin producing Bt isolates. **a** Parasporin reference strain A1470. **b**, **c** Bt isolates T98 and T186, respectively. *c* crystal, *s* spore. *Scale bar* 20 μm

Morphology of the parasporal inclusion bodies of Bt was reported to be heterogeneous (Ohba et al. 2001). Crystal morphology of Bt isolates are of cuboidal, spherical, rhomboidal, and irregular shapes (Bernhard et al. 1997). However, four distinct crystal morphologies are apparent; the bipyramidal crystals are related to Cry1 proteins (Aronson and Fritz-James 1976), cuboidal inclusions related to Cry2 proteins and usually associated with bipyramidal crystals (Ohba and Aizawa 1986); square crystals related to Cry3 proteins (Herrnstand et al. 1986; Lopez-Meza and Ibarra 1996); amorphous and composite crystals related to Cry4 and Cyt proteins (Federici et al. 1990). There is a striking correlation between shape of crystal and spectrum of toxicity (Chambers et al. 1991; Ramalakshmi and Udayasuriyan 2010). Recent reports show parasporin protein inclusions which do not have insecticidal properties also exhibit variation in crystal morphology. The crystal morphology of parasporin producers varies from spherical, bipyramidal to irregular (Kitada et al. 2006). In the present study, four new isolates (T6, T68, T165, and T461) showed spherical inclusions. The isolates T37, T98, and T186 showed irregular-shaped inclusions (Table 2). The crystal shape of the isolates T98 and T186 is similar to the reference strain of *ps4,* A1470 (Fig. 1). Variation in crystal morphology may indicate the diversity of crystal proteins in isolates (Schnepf et al. 1998; Rampersad and Ammons 2005; Ibarra et al. 2003).

**Fig. 2:**
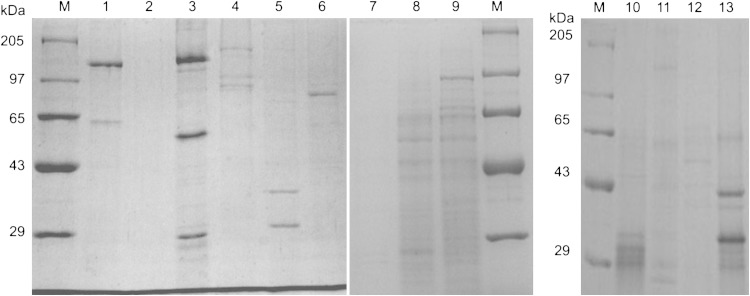
SDS-PAGE analysis of spore–crystal mixture of Bt strains. *M* Genei Protein marker (Higher Range #105977). *Lanes 1–2* reference strain HD1 and 4Q7. *Lanes 3–6* reference strains of parasporin (PS4, PS3, PS2 and PS1). *Lanes 7–13* Bt isolates, T6, T37, T68, T461, T165, T186 and T98

Grouping of Bt isolates according to crystal protein(s) profile analyzed through SDS-PAGE will give a prelude for the presence of diversity in *cry* and *ps* genes. Crystal protein profile of the seven new isolates, reference strains for Cry1, Cry2 (HD1), reference strains for PS1–PS4 (A1190, A1547, A1462, and A1470) and the acrystalliferous reference strain (4Q7) were compared. The reference strain HD1 showed a prominent 135-kDa protein of *cry1* gene and 65-kDa protein of *cry2*. The Bt strain HD1 was included as one of the reference strains, even though it is known to be toxic to lepidopteran insects (Hofte and Whiteley 1989), to observe whether the new isolates produced distinct protein similar to that of HD1 or not. The acrystalliferous reference strain of Bt 4Q7 did not show prominent protein bands as reported earlier (Schnepf et al. 1985; Adang et al. 1985; Widner and Whiteley 1989). The reference strains of *ps1, ps2, ps3*, and *ps4* showed various sized proteins ranging from 29 to 140 kDa as reported earlier workers (Mizuki et al. 2000; Kim et al. 2000; Yamashita et al. 2005; Okumura et al. 2004). All the new isolates had different protein profile when compared to reference strains (Fig. 2). Protein profile of the test isolates T68, T461, and T98 showed prominent multiple bands, whereas the isolates T37, T165, and T186 showed faint multiple bands. The new isolate T6 did not show any prominent protein band. All the new isolates and reference strains of Bt differed from each other suggesting that the parasporin and crystal proteins of the new isolates could be the novel one.

**Fig. 3:**
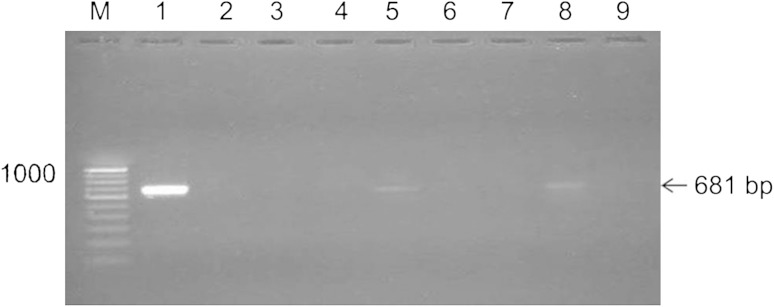
Amplification of *ps4* gene from the test isolates of Bt. *M* 100 bp ladder. *Lane 1* Reference strain of ps4 A1470. *Lanes 2–8* Bt isolates, T6, T37, T68, T461, T165, T186 and T98. *Lanes 9* water control

Among several methods available for characterization of Bt strains, such as PCR, RFLP, Southern blot analysis, and bioassay (Kronstad and Whiteley 1986), PCR is rapid and highly sensitive method for detecting and identifying novel Bt genes. Carrozi et al. (1991) proposed PCR as an accurate and rapid method for identification of novel strains with unknown crystal producing genes. The efficacy of PCR for *cry* genes and *ps* genes identification relies on the alternation of conserved and variable nucleotide regions. All the seven new isolates of Bt were screened for the presence of *cry* genes (*cry1* and *cry2*) and *ps* genes (*ps1*, *ps2*, *ps3,* and *ps4*) by PCR. Primers specific for *cry1* and *cry2* family genes gave amplification of expected size in the reference strain HD1 only. None of the seven new isolates gave amplification to both these gene families, indicating the absence of *cry1* and *cry2* family genes. Primers specific for *ps1, ps2,* and *ps3* genes gave amplification of expected sizes in the respective reference strains of Bt only. Primers specific to *ps4* gene gave amplification of expected size in the reference strain (A1470) and two new isolates, T98 and T186 (Table 3; Fig. 3). This result suggested the presence of *ps4* gene(s) in the two new isolates.

**Table 3:** PCR screening of new isolates of Bt for *cry* and *ps* genes

Isolate	*cry1*	*cry2*	*ps1*	*ps2*	*ps3*	*ps4*
T6	–	–	*	*	–	–
T37	–	–	–	*	–	–
T68	–	–	–	–	–	–
T98	–	–	*	–	–	**+**
T165	–	–	–	–	–	–
T186	–	–	–	*	*	**+**
T461	–	–	–	–	–	–

+ Present, − absent

* Unexpected size

**Fig. 4:**
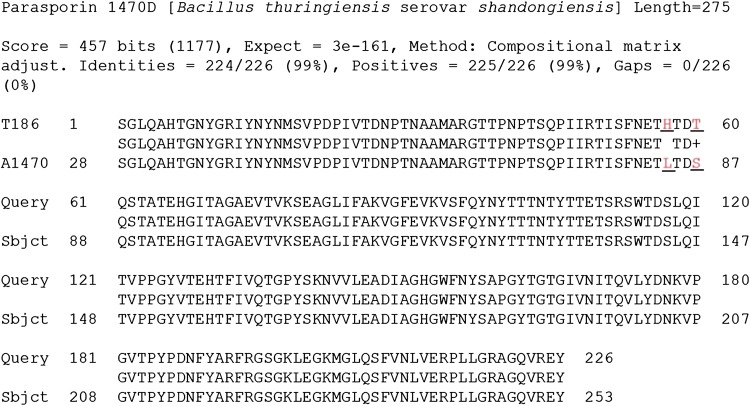
Comparison of deduced amino acid sequence of T186 and Ps4Aa1

The partial *ps4* gene (681 bp) fragment amplified by gene-specific primers from the new isolates T98 and T186 were cloned into pTZ57R/T (T/A) cloning vector. The transformants of *E. coli* were screened by PCR. The nucleotide sequence from the positive clone was generated from Eurofins Genomics India Pvt. Ltd., Bangalore. Sequence similarity analysis of nucleotide sequences of the partial *ps4* gene (681 bp) cloned from the new isolates T98 (KC832499) and T186 (KC832500) with that of *ps4Aa1* showed 100 and 99 % homology, respectively. Comparison of deduced amino acid sequence of T186 with that of *ps4Aa1* showed variation in two positions. At position 84, leucine is replaced by histidine, and at position 87 serine by threonine (Fig. 4). Thus, the sequence of partial *ps4Aa* gene cloned from the two new isolates showed high homology with its holotype *ps4Aa1*. It confirms the presence of *ps4Aa* type gene in the two isolates, T98 and T186.

**Fig. 5:**
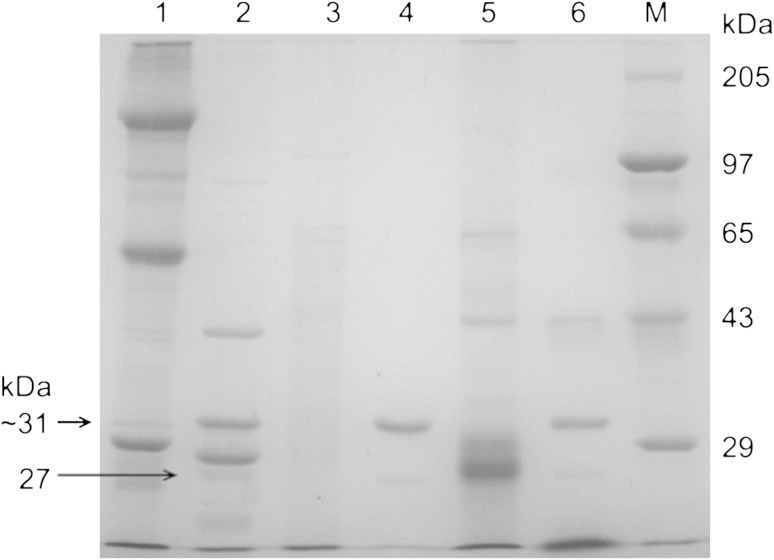
Proteolytic processing of inclusion proteins of Bt strains. *M* Genei Protein marker. *Lanes 1, 3, 5* solublized inclusion protein. *Lanes 2, 4, 6* proteinase K-treated solubilized protein. *Lanes 1, 2* reference strain of ps4 A1470. *Lanes 3 and 4, 5 and 6* Bt isolates T186 and T98, respectively

A study on proteolytic processing of crystal proteins from the *ps4* harboring isolates T98, T186, and reference strain of *ps*4 (A1470) by SDS-PAGE, showed a major polypeptide of 40-kDa; two prominent bands: one at >29 kDa and another at <29 kDa; and a faint band at 27-kDa in the solubilized protein of reference strain of *ps4* (Fig. 5) as reported earlier (Saitoh et al. 2006). Similar to that of the *ps4* reference strain, the proteinase K-treated protein of new isolates (T98, T186) also showed a faint band at 27-kDa and a prominent band of >29 kDa (31-kDa). The protein of PS4 and the proteinase K are of same molecular weight ~31 kDa (Saitoh et al. 2006). Hence the prominent band at 31-kDa in proteolytic processed samples corresponds to proteinase K. As reported earlier, 31-kDa protoxin of PS4 will be digested to a 27-kDa toxin upon proteolytic processing. Therefore, it can be suggested that the faint band of 27-kDa polypeptide in the isolates (T98 and T186) may be proteolytic product of the 31 kDa PS4 protein. This gives the evidence that the test isolates may be parasporin producers. In addition, a prominent band of 43-kDa is also seen in the isolate T98 which discriminates the isolate from T186.

Generally, the parasporin protein producing strains of Bt do not produce any insecticidal protein (Kitada et al. 2006; Mizuki et al. 1999a). The two new isolates of the present study, T98 and T186 (which showed presence of *ps4* gene) did not show toxicity on *S. litura* and *H. armigera.* Growth inhibition of insect larvae was also not observed in both the new isolates. The reference strains A1470 and 4Q7 also recorded the same results; whereas, the reference strain of *cry1* and *cry2* genes (HD1) showed 100 % mortality on both *S. litura* and *H. armigera.* Mizuki et al. ( 1999a) also reported that PS4 producers do not have insecticidal activity on lepidopteran (*Plutella xylostella* and *Bombyx mori*) and dipteran pests (*Aedes aegypti, Culex pipiens molestus, Anopheles stephensi, Telmatoscopus albipunctatus,* and *Musca domestica*).

## Conclusion

Based on protein profile, PCR screening, nucleotide sequencing and insect bioassay, it is evident that the parasporin producing strains are members in *B. thuringiensis* populations occurring in natural environments of India. Cloning and characterization of complete gene (*ps4*) and evaluation of these isolates for their anticancer properties are required for identifying potential use of parasporin proteins in anticancer medical research. 
